# Deregulation of Hepatic Insulin Sensitivity Induced by Central Lipid Infusion in Rats Is Mediated by Nitric Oxide

**DOI:** 10.1371/journal.pone.0006649

**Published:** 2009-08-14

**Authors:** Nicolas Marsollier, Nadim Kassis, Karima Mezghenna, Maud Soty, Xavier Fioramonti, Amélie Lacombe, Aurélie Joly, Bruno Pillot, Carine Zitoun, José Vilar, Gilles Mithieux, René Gross, Anne-Dominique Lajoix, Vanessa Routh, Christophe Magnan, Céline Cruciani-Guglielmacci

**Affiliations:** 1 CNRS-Université Paris Diderot, Paris, France; 2 CNRS UMR 5232, Faculté de Pharmacie, Montpellier, France; 3 INSERM, U855, Lyon, France; 4 Université de Lyon, U1235, Lyon, France; 5 Universite Claude Bernard Lyon I, Villeurbanne, France; 6 Department of Pharmacology and Physiology, New Jersey Medical School, Newark, New Jersey, United States of America; 7 INSERM U689, Hôpital Lariboisière, Paris, France; University of Camerino, Italy

## Abstract

**Background:**

Deregulation of hypothalamic fatty acid sensing lead to hepatic insulin-resistance which may partly contribute to further impairment of glucose homeostasis.

**Methodology:**

We investigated here whether hypothalamic nitric oxide (NO) could mediate deleterious peripheral effect of central lipid overload. Thus we infused rats for 24 hours into carotid artery towards brain, either with heparinized triglyceride emulsion (Intralipid, IL) or heparinized saline (control rats).

**Principal Findings:**

Lipids infusion led to hepatic insulin-resistance partly related to a decreased parasympathetic activity in the liver assessed by an increased acetylcholinesterase activity. Hypothalamic nitric oxide synthases (NOS) activities were significantly increased in IL rats, as the catalytically active neuronal NOS (nNOS) dimers compared to controls. This was related to a decrease in expression of protein inhibitor of nNOS (PIN). Effect of IL infusion on deregulated hepatic insulin-sensitivity was reversed by carotid injection of non selective NOS inhibitor NG-monomethyl-L-arginine (L-NMMA) and also by a selective inhibitor of the nNOS isoform, 7-Nitro-Indazole (7-Ni). In addition, NO donor injection (L-arginine and SNP) within carotid in control rats mimicked lipid effects onto impaired hepatic insulin sensitivity. In parallel we showed that cultured VMH neurons produce NO in response to fatty acid (oleic acid).

**Conclusions/Significance:**

We conclude that cerebral fatty acid overload induces an enhancement of nNOS activity within hypothalamus which is, at least in part, responsible fatty acid increased hepatic glucose production.

## Introduction

Metabolic Syndrome is a constellation of disturbance where insulin resistance is considered as a key factor in the onset of disorders [1]. In the early stage, and before any change in fat mass and/or ectopic storage of triglycerides (TG) within liver or muscle, changes in autonomic nervous system (ANS) activity could modify both insulin secretion and action [2]–[4]. In central nervous system (CNS), hypothalamus is a key regulator of ANS output as well as a nutrient sensor. Indeed, growing evidences show involvement of both glucose [5]–[7] and fatty acids (FA) [8], [9] sensitive neurons within hypothalamus, especially arcuate and ventromedial part, in the regulation of both energy homeostasis and food intake [10]–[12]. It has been shown that short term intracerebroventricular infusion of oleic acid reduced both food intake and hepatic glucose production [13]. Besides this acute effect of hypothalamic FA sensing, it appeared that central FA overload -which could occur during metabolic diseases- deregulate such sensing, thus leading to impaired CNS control of glucose homeostasis, insulin secretion and sensitivity [14]. This deregulation of sensing may impair CNS control of hepatic glucose output through dysfunction of autonomic balance and hypothalamic ANS output [15], [16]. However, the molecular events involved in effects of high FA exposure in the hypothalamus are still poorly understood. In the present study, we hypothesized that the hypothalamic nitric oxide (NO) production mediates the central effects of FA. Indeed it has been shown in other tissues such as pancreatic beta cell that lipids exposure activates NO pathway in physiological and physiopathological conditions [17], [18]. NO is generated from L-Arginine amino acid by NO synthase (NOS), a process that occurs in most tissues including brain [19]. There are 3 different isoforms of NOS which can be divided in two categories: constitutive, Ca^2+^ dependent NOS (endothelial or eNOS, and neuronal or nNOS), and inducible NOS (iNOS), Ca^2+^ independent. Constitutive NOS mediates cell signaling, whereas iNOS activation occurs during inflammation, and leads to cytotoxic effects. Since iNOS produces larger amount of NO than constitutive NOS [20], NO generation by the latter appears to be more adapted in cell signaling. nNOS activity is partly regulated by PIN (protein inhibitor of neuronal NOS), which has been identified in rat brain [21]. Binding of PIN prevents homodimerization of nNOS with as a result an inhibition of NO production (14). Recently NO has been identified as a regulator of nutrient metabolism [22] and its production is modulated by dietary factors [23]. NO is also a modulator of electrical activity of hypothalamic neurons involved in the control of energy homeostasis [24], [25].

We hypothesized that central lipid overload may induce NO production within the hypothalamus which in turn may lead to impairment of insulin secretion and action through changes in autonomic nervous system output.

## Results

### Oleic acid stimulates NO production in rat ventromedial hypothalamic (VMH) neurons (Figure 1)

**Figure 1:**
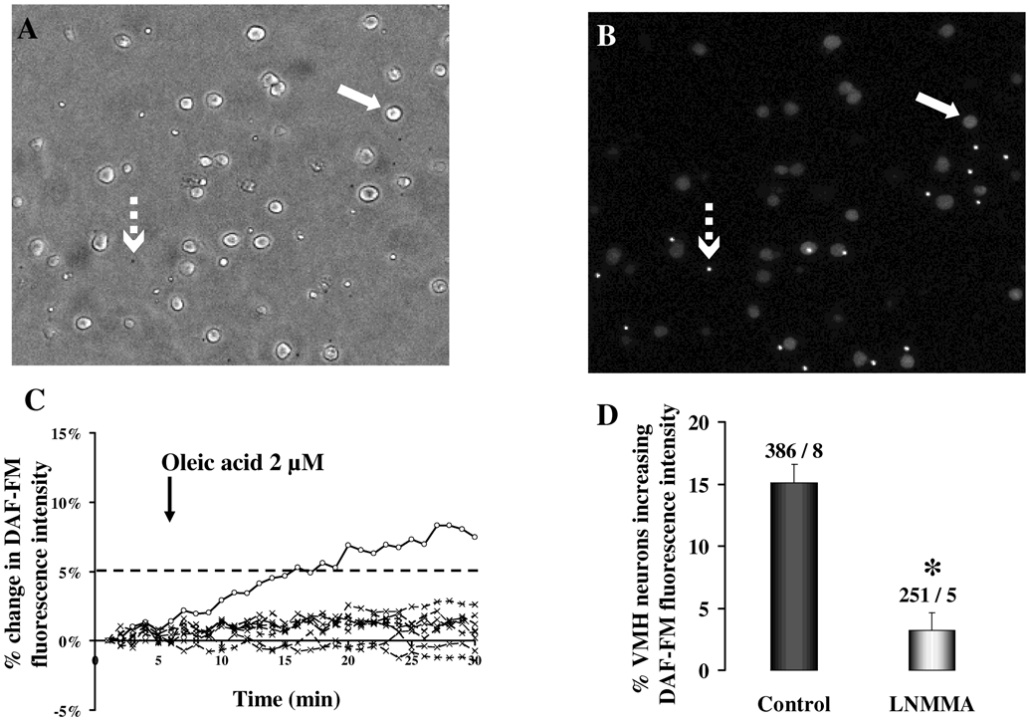
NO production by VMH neurons in response to oleic acid. A and B: Bright field (A) and DAF-FM fluorescence intensity images of cultured (24 H) VMH neurons from rats. Cells (solid arrow) are plated with fluorescent beads (broken arrow) which are used to calibrate changes in cell fluorescence intensity. C: Example of VMH neurons which increase (solid line, 1 neuron) or do not change (dashed line, 8 neurons) their DAF-FM fluorescence intensity in response to oleic acid (2 µM). Neurons increasing their DAF-FM fluorescence intensity above 5% after 15–25 min post oleic acid addition are considered to produce NO in response to oleic acid. D: Percentage of VMH neurons that increased their DAF-FM fluorescence intensity [NO production] in response to oleic acid (2 µM) in the presence or absence (Control) of the non-selective NOS inhibitor N-Methyl-L-arginine (L-NMMA, 1 mM). Data are presented as mean±sem. *: p<0.05. Total number of cells – number of dishes used are given on the top of each bar.

DAF-FM loaded VMH cultured neurons were visualized under brightfield and fluorescent (FITC) microscopy (Figure 1A, 1B). DAF-FM fluorescence intensity increased when NO was produced. Figure 1C shows an example of a group of neurons where one of them increased its DAF-FM fluorescence intensity above 5% and eight of them did not increase their DAF-FM intensity in response to 2 µM oleic acid. Overall, oleic acid increased DAF-FM fluorescence intensity in 15.1±1.53% of VMH cultured neurons (n = 386; Figure 1D). Non selective NOS inhibitor L-NMMA significantly decreased the DAF-FM intensity in response to oleic acid to 3.23±1.4% (n = 251; Figure 1D). We conclude that some VMH neurons respond to fatty acid by producing NO.

### Plasma parameters and food intake following intracarotid IL infusion (Table 1)

Neither plasma insulin nor glucose concentrations were modified in IL rats compared with C rats at the end of the 24 h lipid infusion. Plasma FA concentration is not modified by lipid/heparin infusion (Time course C vs. IL: 0 h: 419.1±43.4 µM vs. 419±57,1 µM ; 4 h : 543.3±71.5 µM vs. 542.6±50.1 µM ; 8 h : 559.1±57.2 µM vs. 544.6±50.2 µM; 20 h: 504.3±45.6 µM vs. 528.5±22.4 µM; 24 h: 523.6±80.5 µM vs. 499.3±3.5 µM). Plasma corticosterone concentration was significantly decreased in IL rats whereas plasma ACTH concentration was unchanged. IL/heparin infusion had no effect on body weight or food intake (Table 1).

**Table 1:** Basal characteristics of the experimental groups after 24 h of carotid infusion.

	C	IL
**Body weight (g)**	236.7±7.7	235.1±9.7
**Food intake (g/Kg bw)**	79.85±4.3	73.4±5
**Plasma TG (mg/ml)**	0.81±0.09	0.75±0.09
**Glycemia (mmol/l)**	6.6±0.15	6.55±0.14
**Insulinemia (pmol/l)**	288±36.3	246.5±20.7
**Corticosteronemia (ng/ml)**	416±38.6	252.9±43.9*
**Plasma ACTH (pg/ml)**	265±38	207.1±28.4

C, control rats infused with saline/heparin for 24 h through the carotid artery; IL, rats infused with Intralipid/heparin for 24 h through the carotid artery. Values are means±SEM in each group.

* p<0.05 compared to C rats.

### Intracarotid IL infusion increases hypothalamic NOS activities (Figure 2)

**Figure 2:**
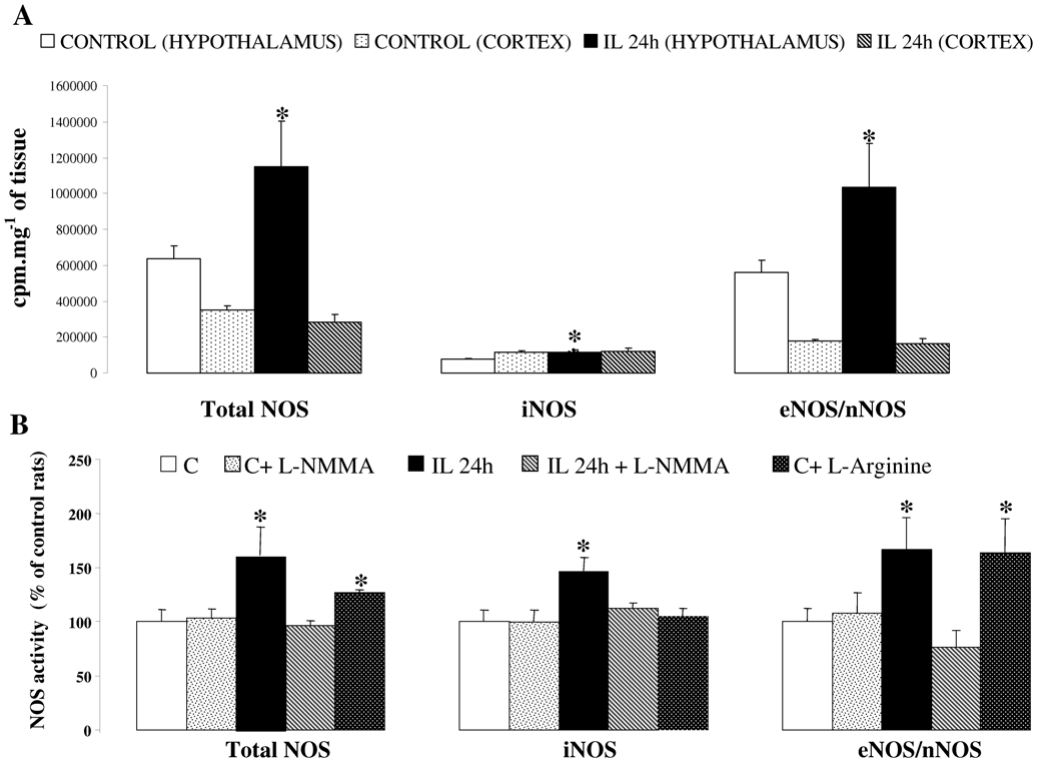
Effect of lipid infusion on central NOS activities. A: Hypothalamic and cortical NOS activities after 24 h of Intralipid/heparin infusion (IL 24 h) or saline/heparin infusion control group (C). Results are expressed as c.p.m per mg of tissue. B: Hypothalamic NOS activities of IL and C rats treated or not with acute injection of non selective NOS inhibitor, L-NMMA. The last right bar displays NOS activities in C rats injected with NOS substrate, L-Arginine. Results are expressed as percentage of C group activities. * p<0.05 vs. C.

In the IL group, the activity of total NOS and constitutive (nNOS/eNOS) was increased in mediobasal hypothalamus (respectively by 81% and 84%) compared to C rats. Moreover cortical NOS activity was not modified by infusion (Figure 2A). Carotid injection of L-NMMA (non selective NOS inhibitor) normalized NOS activities in IL rats without any change in C rats. L-Arginine administration stimulated constitutive NOS activity in C rats at the enzyme activity reached the same level as in the IL group (Figure 2B). iNOS activity was significantly increased in IL hypothalamus, but not in cortex (Figure 2A/B).

Systolic blood pressure measurement reveal that lipid infusion or L-NMMA injection into carotid catheter, in C rats as well as in IL rats, did not lead to significant changes in vascular tone and heart beat, compared to a control saline injection. (data not shown)

### Effect of intracarotid IL infusion on liver parameters in basal state (Figure 3)

**Figure 3:**
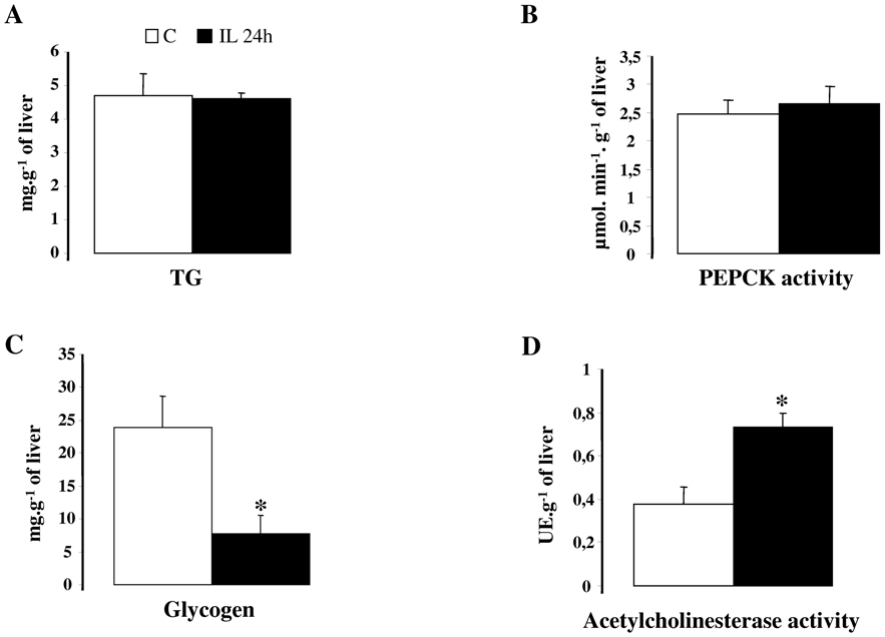
Hepatic parameters after 24 h of infusion in C rats (NaCl/heparin) and in IL rats (IL/hep). A: liver triglycerides content, B: liver PEPCK activity, C: liver glycogen, D: liver acetylcholinesterase activity. Values are means±SEM in each group. * p<0.05 vs. controls.

Liver TG content (Figure 3A) and PEPCK activity (Figure 3B) are not modified in IL rats, whereas the hepatic glycogen content was significantly reduced in IL rats (Figure 3C). IL rats liver cholinesterase activity was 2 fold increased compared to controls (Fig. 3D).

### Central NOS inhibitors and NO donors regulate glucose-induced insulin secretion (Figure 4)

**Figure 4:**
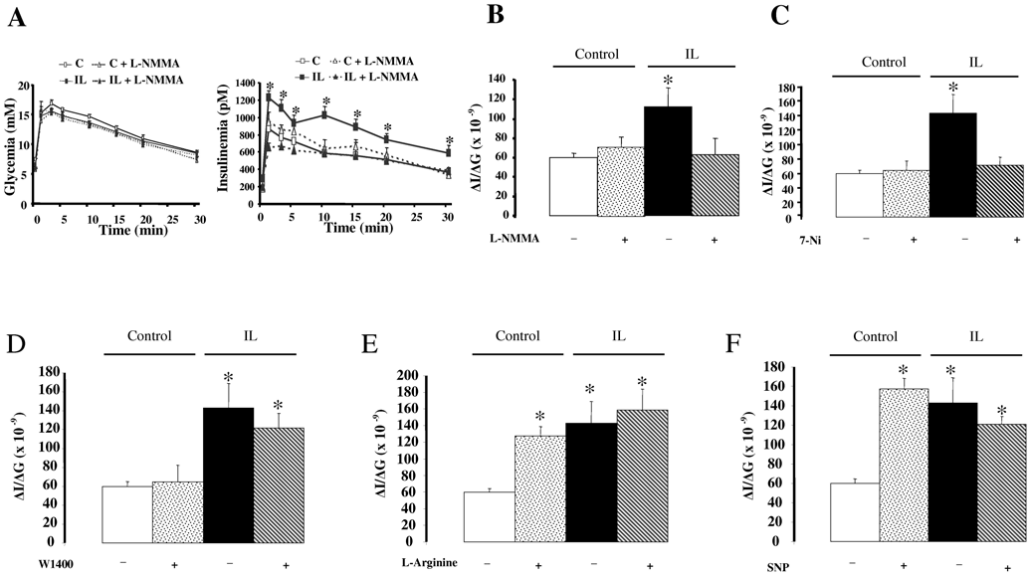
Assessment of glucose induced insulin secretion. Time course of plasma glucose and insulin (A) concentrations in response to glucose load in C rats (open squares), C+L-NMMA rats (open triangles, dotted line), IL rats (filled squares) and IL+L-NMMA rats (filled triangles, dotted line). B, C, D, E, F: insulinogenic index (ΔI/ΔG), i.e. the area under curve of insulinemia time course divided by the area under curve of glycemia time course, after carotid injections of respectively with L-NMMA, 7-Ni, W1400, L-Arginine and SNP. Drugs or saline are injected 5 minutes before glucose loading into saphenous vein. Values are means±SEM in each group. * p<0.01 vs C rats. L-NMMA: non selective NOS inhibitor. 7-Ni: selective neuronal NOS inhibitor. W1400: selective inflammatory NOS inhibitor. SNP: chemical NO donor. IL 24 h: rats infused with of Intralipid/heparin through the carotid artery towards the brain for 24 h. C: rats infused for 24 h with saline/heparin.

The time course of glycemia following glucose loading was similar in control rats and IL rats, independently to carotid injection of non selective NOS inhibitor L-NMMA (Figure 4A). In contrast, glucose-induced insulin secretion (GIIS) was greater in IL rats than in C rats (time-course of insulinemia (Figure 4A) and insulinogenic index, ΔI/ΔG, Figure 4B). L-NMMA injection reversed this hypersecretion of insulin showed in IL rats whereas it had no significant effect on GIIS in control rats (Figure 4A/B). Selective nNOS inhibitor 7-Ni treatment through carotid artery also normalized ΔI/ΔG in IL rats (Figure 4C). In contrast injection of W1400 (selective iNOS inhibitor) did not reversed increased GIIS in IL rats (Figure 4D). Treatments with L-Arginine (NOS substrate) and SNP (chemical NO donor) significantly increased ΔI/ΔG in C rats, to reach a value similar to that of IL rats (Figures 4E and 4F), mainly through an increase in insulin plasma concentration.

### Central NOS inhibitors and NO donor effects on glucose turnover rate (Figures 5)

**Figure 5:**
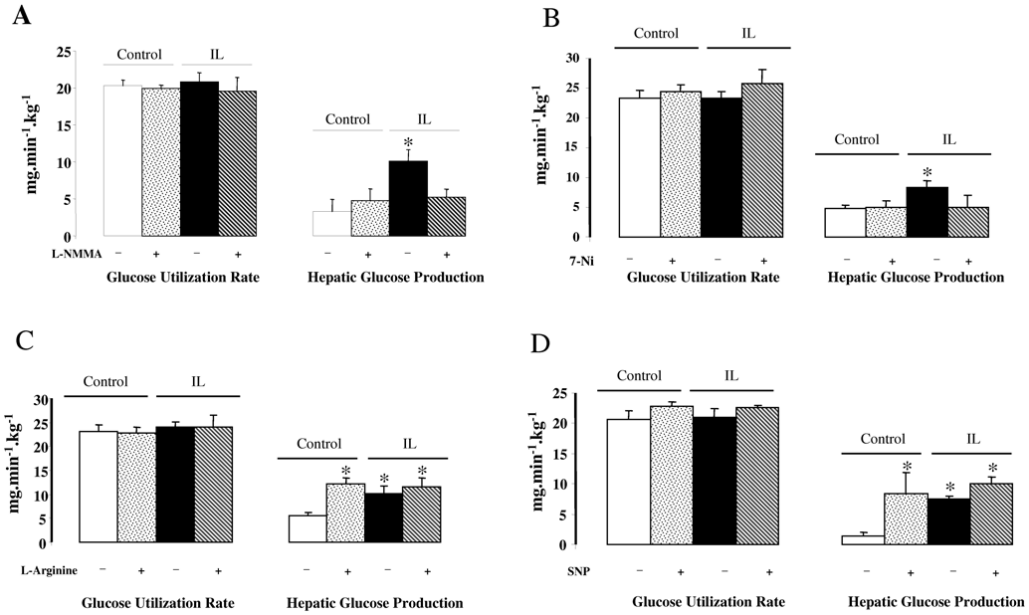
Glucose turnover rate during euglycemic-hyperinsulinemic clamps. Glucose Utilization and Hepatic Glucose Production during euglycemic-hyperinsulinemic clamp (0.4 U·kg^−1^·h^−1^) in Control and IL rats before and after L-NMMA (A), 7-Ni (B). L-Arginine (C) or SNP (E) carotid injection. L-NMMA: non selective NOS inhibitor. 7-Ni: selective neuronal NOS inhibitor. W1400: selective inflammatory NOS inhibitor. SNP: chemical NO donor. Values are means±SEM in each group. * p<0.01 vs C rats.

Glucose turnover rate (GTR) was measured in basal and under hyperinsulinemic-euglycemic clamp conditions. In basal state GTR was similar in both groups (∼10 mg/min/Kg, not shown). During hyperinsulinemic-euglycemic clamp procedure, plasma insulin concentrations were increased to a similar extent (∼3.5-fold above basal values) in all groups. Glucose infusion rate (GIR) at the steady-state was significantly lower in IL rats (14.5±1.1 *vs*. 21.83±1.5, p<0.01) whereas increase in glucose utilization was similar in C and IL rats (Figure 5A). In contrast hepatic glucose production (HGP) remained significantly higher (*p*<0.01) in IL rats than C rats, highlighting hepatic insulin resistance (Figure 5A).

Carotid injections of L-NMMA had no effect on HGP in C rats but strongly decreased hepatic glucose output in IL rats, thus normalizing hepatic insulin sensitivity (Figure 5A). As selective iNOS inhibitor (W1400) had no effect on GIIS, the drug was not tested in the clamp study. Regarding effect of nNOS selective inhibitor (7-Ni), HGP was normalized in IL rats (Figure 5B). This suggests that inhibition of nNOS is sufficient to counteract effect of IL infusion on glucose homeostasis.

The carotid injection of NOS substrate, L-Arginine, as well as SNP (chemical NO donor) led to an increase in HGP in C rat which reflected the induction of hepatic insulin resistance (Figure 5C and 5D).

### Liver parameters during clamp procedure (Figure 6)

**Figure 6:**
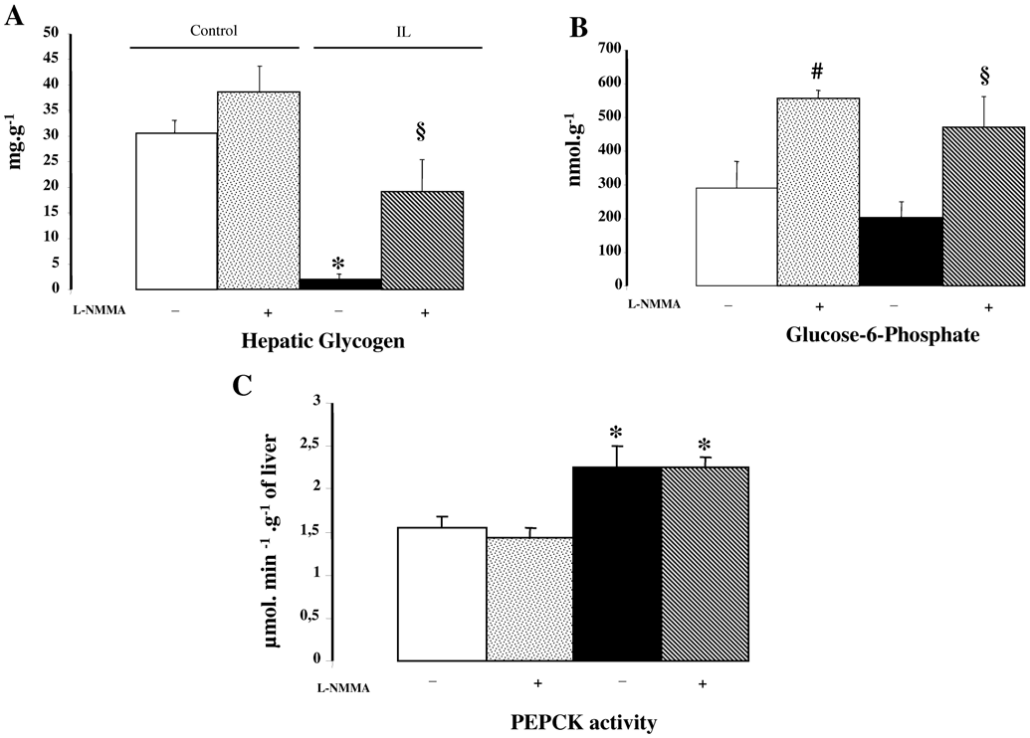
Hepatic parameters during euglycemic-hyperinsulinemic clamp procedure. A: Glycogen content, B: Glucose-6-Phosphate content, C: PEPCK activity in the liver of control and IL rats (injected or not with non selective NOS inhibitor, L-NMMA) at the end of the clamp procedure. Values are means±SEM in each group. * p<0.01 vs. C rats; § p<0.01 vs. IL rats.

As shown in Figure 6A, at the end of the clamp procedure, the glycogen content was markedly reduced in IL rats compared to C rats, and both values were similar to those measured in basal condition (see Figure 3C). Carotid injection of L-NMMA (non selective NOS inhibitor) partially restored glycogen content of IL rats, whereas no significant changes were seen in C rats. L-NMMA injection led to a 2-fold increase in G-6-P content in both IL and C rats (Figure 6B). Liver PEPCK activity was more elevated in IL rats, thus reflecting increased gluconeogenesis, and L-NMMA injection did not affect it (Figure 6C).

### Hypothalamic nNOS dimer/monomer ratio is increased by IL infusion (Figure 7)

**Figure 7:**
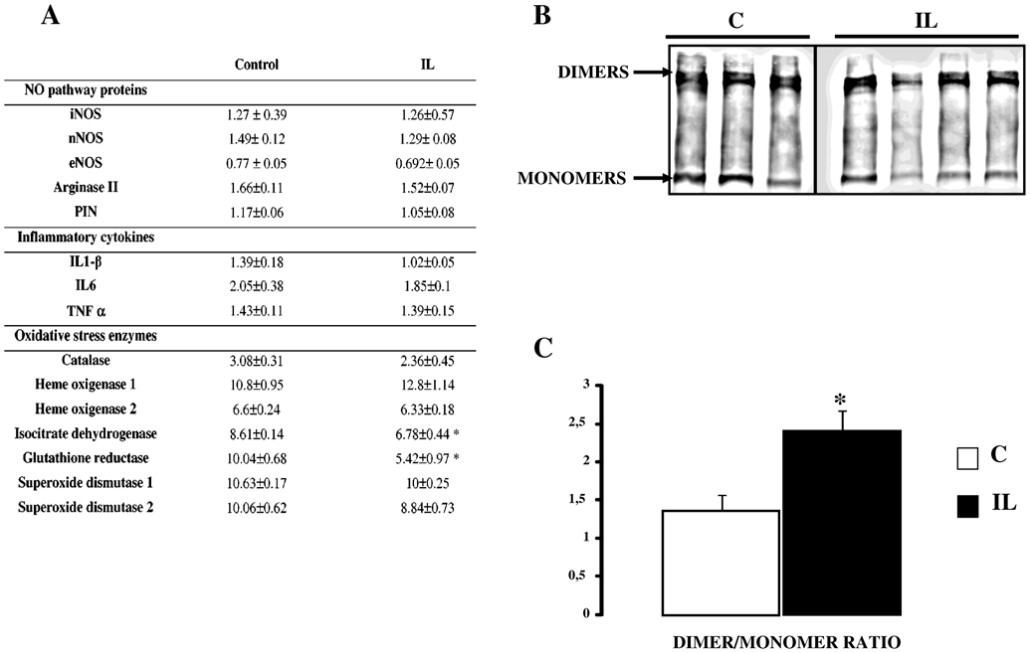
Hypothalamic gene expression after 24 h IL infusion. A: mRNA expression of NO pathway proteins, inflammatory cytokines and oxidative stress enzymes in the hypothalamus of C rats and IL rats. Values are means±SEM in each group (arbitrary units). B: Western blot of nNOS monomers and dimers in C and IL rats, and D: expression of the ratio dimers/monomers (D/M) after image analysis (quantification of the intensity of the blots). Values are means±SEM in each group. * p<0.01 vs C rats.

The mRNA levels of the three NOS in the hypothalamus of IL rats did not differ from controls (Figure 7A). However, as shown in Figures 7B and 7C, 24 h of lipid/heparin infusion led to a marked increase in the dimer/monomer ratio of the nNOS isoform, which could explain the increased activities we measured. In addition, PIN mRNA level appeared unchanged (Figure 7A), whereas the PIN protein showed a tendency to decrease in IL rats (9.6±2.2 vs 12.32±3 in C rats, NS). No significant change in gene expression were observed for the inflammatory cytokines (Figure 7A), known to be involved in iNOS induction [20]. Likewise, oxidative stress enzymes, which could interfere with NO metabolism, remained unchanged with the following two exceptions. The expression of isocitrate dehydrogenase and glutathione reductase, enzymes known to be inactivated by NO [26], [27], were significantly down-regulated in the mediobasal hypothalamus of IL rats (Figure 7A). Moreover IL rats displayed a 45% decrease in glutathione reductase enzymatic activity in the hypothalamus, compared to controls (data not shown).

## Discussion

In this study we showed that central lipid overload led to increased hypothalamic NOS activity and consequently to both hepatic insulin resistance and increased GIIS. Effects of lipid overload were mimicked in normal rats centrally receiving NO donors and selective inhibition of hypothalamic nNOS activity was sufficient to block such effects.

First of all, we demonstrated that central lipid overload increased hypothalamic NOS activities. We cannot totally exclude an extra-hypothalamic effect; however we reported here that no change in NOS activities was observed in cortex of IL infused rats compared to controls. In addition, the structure and function of blood brain barrier (BBB) within hypothalamus -especially median eminence- is quite different from other part of the brain (review in [28]) and Norsted et al recently evidenced that in medio-basal hypothalamus the structure of the endothelium is not composed by the same proportion of occludins and tight junctions suggesting an increased permeability to circulating substances [29]. Finally, it has been demonstrated that palmitate uptake in hypothalamus is about 10 to 15% versus less than 2% in other brain areas [30]. Taken together, these data suggest a local effect of lipids close to their route of infusion.

As a basis for the *in vivo* study, we demonstrated that oleic acid stimulated NOS-dependent NO production in cultured VMH neurons (a nucleus in which NOS-immunostaining has been also described [31] and known to be involved in the regulation of both energy homeostasis and autonomic nerves output). It does not rule out that glial/endothelial cells may also play a role in vivo [32], and we cannot exclude that NO production may also occur in other hypothalamic nuclei. Indeed we previously reported that c-fos immunostaining, a marker of neuronal activation, was induced in arcuate nucleus as well as paraventricular nucleus in response to IL infusion [14]. It must be pointed out that c-fos immunostaining was not changed in other part of the brain in IL *vs*. control rats [14].

In the present study we show that 24 h lipid-infusion increase hypothalamic NOS activity and this is mainly due to constitutive NOS isoforms activation. While an increase in the three NOS isoforms activities occurred, iNOS activation was probably not a key actor. First, increased iNOS activity was modest, as depicted in figure 2, compared to constitutive NOS. In addition, this was not associated with increased mRNA expression of enzyme and inflammatory cytokines, as classically observed in iNOS pathway activation [20]. Finally, to further identify which NOS isoform was responsible for IL effects, pharmacological studies were performed and pretreatment with a selective iNOS inhibitor (W1400) did not prevent lipid effects on GIIS. Taken together, iNOS activity implication in IL effects is probably modest compared to constitutive NOS. However, pretreatment with L-NMMA, a non selective NOS inhibitor, prevented lipids effects. To further determine which isoform was mainly involved we made additional studies with selective NOS inhibitors and we observed that pretreatment with the nNOS selective inhibitor, 7-Ni, was sufficient to normalize insulin secretion and action in liver, suggesting a key role for this isoform in mediating lipid effects. However, since activity assay does not discriminate between eNOS and nNOS, we cannot totally exclude that increased eNOS activity –if it occurred- could be also partly responsible for IL effect.

Some hypothesis can be made to elucidate the way of action of NO. From our *in vitro* study, it appears that a rapid NO production could also imply a rapid action on neuronal membrane potential. It has been reported in isolated islets of Langerhans that NO may act through GPR40 by modulating intracellular calcium concentration [33]. In addition, several studies suggested that NMDA-evoked intracellular [Ca^2+^] transients were modulated by endogenous NO production, which lead to release of calcium from the mitochondrial pool [34], or that NO synthesis was important for the proaversive effects produced by activation of glutamate-NMDA receptors located within limbic midbrain structure [35]. Thus effect of NO could involve change in channel activities and consequently modulation of neuronal activities. Besides this effect of NO, we previously reported that fatty acid metabolism was important to modulate their effects [14] suggesting involvement of several pathways.

Our data also showed that lipid-induced nNOS activity could be related to the increase of nNOS dimer to monomer ratio, probably ensue from the diminution of PIN expression, although non significant. Indeed, this could account for the increased NO production [36]. This modulation of nNOS catalytic activity is assume to prevent deleterious NO overproduction as reported in a study where PIN expression was increased [37]. In addition, acute injection in control rats with either NOS substrate L-arginine, or chemical NO donor, SNP, led to hepatic insulin resistance and increased GIIS, strengthening the idea that enhanced hypothalamic NO production was an essential step in the onset of these metabolic dysfunctions.

Activity and mRNA expression of glutathione reductase, a potent anti-oxidant enzyme, was also decreased in the hypothalamus of IL rats. This could also contribute to an increase in cellular NO availability independently of an action on NOS activity [38]. Taking these data together, we can devise that a context of lipid excess may lead to both enhancement of hypothalamic NOS activity and NO availability.

Increased NOS activities within hypothalamus following 24 h of carotid lipid infusion led to hepatic insulin resistance. This is consistent with previously described effects, demonstrating that high levels of lipid exposure in the hypothalamus may partly contribute to the etiology of type 2 diabetes [39], [40]. In our model this hepatic insulin-resistance was not related to ectopic storage of TG within liver. Thus a major impact of carotid IL infusion could be related to change in the ANS output, especially parasympathetic component. Indeed, as attested in this study, IL rats displayed a 2-fold increase in liver acetylcholinesterase activity, which is likely to promote increased breakdown of acetylcholine and reduced activity at the muscarinic receptor level [41]. Since the parasympathetic nervous system stimulates glycogen synthase activity [42], a lower parasympathetic function within the liver could partly explain the decreased glycogen content - hallmark of liver insulin resistance [43], [44]- observed in our IL rats. This has been demonstrated in a model of rats with specific liver vagotomy, which display significant impairment in liver glycogen storage and impaired hepatic sensitivity to insulin [45]. Thus, even if we did not evidenced a clear cause-effect relationship, we can conclude from our data, that there is a correlation between increased cholinesterase activity and decreased hepatic glycogen content. In addition, it has been reported that NO modulates pre-autonomic neurons activity in the paraventricular nucleus of the hypothalamus, a key regulator of ANS output [46], [47]. Finally we could also hypothesize that increased insulin secretion in response to central IL infusion could, at least in part, be responsible for increased hepatic insulin resistance.

As mentioned above, both L-NMMA and 7-Ni treatments prevented the deleterious effects of central lipid overload on hepatic insulin sensitivity. Regarding the activity of the gluconeogenic enzyme PEPCK, elevated in IL rats during EG-HI clamps, L-NMMA had no effect on it. This suggests that hepatic effects of central NOS enhancement mainly involve a control of liver glycogenolysis. Moreover, central L-NMMA injection increased liver glucose-6-phosphate content in both groups, indicating a better insulin sensitivity [43]. Together, with the unchanged PEPCK activity, this suggests that the main point of control is depending on a decreased flux of substrate through glucose-6-phosphatase. In addition, there is only hepatic insulin resistance whereas insulin stimulated glucose uptake remained similar in both groups. Furthermore, increased GIIS in IL rats suggests an adaptation to insulin-resistance.

In conclusion, we demonstrated here that impairment of hepatic insulin action in response to lipid overload within hypothalamus is related to increased NOS activity, mainly involving nNOS. Deregulation of hypothalamic lipid sensing and NO signaling pathway is involved in the setting of hepatic insulin resistance, likely through changes in parasympathetic nervous function, and could take an active part in the early dysfunctions leading to type 2 diabetes in predisposed subjects.

## Materials and Methods

### Animals and experimental design

The experimental protocol was approved by the institutional Animal Care and Use Committee of the University of Paris 7. Male Wistar rats, two-month-old, 250–275 g, (Charles River, l'Arbresle, France) were housed individually in stainless steel cages in a room maintained at 24±3°C with lights on from 7 am to 7 pm hours. Food (regular chow diet: proteins 19.4%; carbohydrates 59.5%; lipids 4.6%; vitamins and minerals 16.5%, Usine d'Alimentation Rationnelle, Rabalot, France) and water was provided *ad libitum*.

### Carotid lipid infusions

The unrestrained infusion technique was used, as previously described [48]. Briefly, before the beginning of the infusion, rats were anaesthetized with pentobarbital (50 mg/Kg i.p.; Sanofi, Libourne, France) for catheterization of right carotid artery, towards the brain. Catheter was then exteriorized at the vertex of the head, and animals were allowed to recover for 5 days. Then catheter is connected to a swiveling infusion device, allowing the animal free access to water and food. Rats were infused with a triglyceride emulsion (Intralipid 20%; KabeVitrum, Stockholm, Sweden) heparinized (20 U/mL) (IL rats), whereas control rats (C rats) were infused with saline/heparin, at 2 µL/min on 24 hours. Heparin was added to the lipid emulsion in order to stimulate lipoprotein lipase activity. Arteriovenous blood was collected from caudal vessels (∼80 µL) for measurement of plasma FA, glucose and hormones concentrations.

### Measurement of NO production in VMH cultured neurons using 4-amino-5-methylamino-2′,7′-difluorofluorescein (DAF-FM)

The experimental protocol was approved by the institutional Animal Care and Use Committee of the University of Medicine and Dentistry of New Jersey. 3–4 weeks old male rats were used in order to prepare VMH dissociated cultured neurons as previously described [24], [25]. Twelve hours after, neurons were incubated with 1 µM DAF-FM at 37°C with extracellular fluid (ECF) containing 2.5 mM of glucose for 30–45 min. DAF-FM is then removed from the cells, and neurons were visualized on an Olympus BX61 WI microscope with a 10X objective for measurement of DAF-FM intensity fluorescence (green filter, excitation 488 nm, emission 515–530 nm). Images are captured (Photometrics, Cool Snap HQ CCD camera) every minutes for 30 min and acquired/analyzed with MetaMorph software (Universal Imaging Corporation). The fluorescence intensity was expressed as gray scale units/pixel. Data were normalized according to the intensity of the fluorescent bead standards coated with the cells on the coverslips. Control images were taken for 5 minutes and oleic acid (2 µM, Sigma Aldrich) was added to the media. At this concentration oleic acid is water soluble. Then the percent change of DAF-FM fluorescence intensity for each neuron was calculated from the control images. As previously showed, neurons increasing their DAF-FM fluorescence intensity above 5% were considered as NO producing cells.

### Food intake

Daily food intake was measured at 9 am. Food intake is reported to body weight of animals and expressed in gram of food per kilo of body weight.

### Liver analysis: TG, glycogen and glucose 6-phosphate (G-6-P) contents, acetylcholinesterase activity

All livers were sampled using the freeze-clamp procedure. The liver TG content was measured with an assay based on glycerol detection [49]. Briefly, 0.1 g of frozen liver tissue was minced in 2 ml of 4°C ethanol. Cell debris was allowed to settle, and further clarification was achieved by centrifugation of the supernatant at 15,000 g for 10 min at room temperature [50]. 10 µL of the supernatant were used to dose TG concentration using the serum Triglyceride Determination kit (Sigma, St. Louis, MO). Liver glycogen and glucose-6-phosphate content were determined according to previously described enzymatic assay procedures [51] [52]. PEPCK activity was assayed in the supernatant of liver homogenates according to the procedure described [53]. Determination of liver acetylcholinesterase activity (Amplex® Red Acetylcholine/Acetylcholinesterase Assay Kit, Molecular Probes, Paisley, UK), was performed to evaluate parasympathetic nervous system function.

### Measurement of hypothalamic NOS activities

At the end of infusion periods, rats underwent an acute carotid injection of non-isoform specific NOS inhibitor, NG-monomethyl-L-arginine (L-NMMA, 0.5 mg/Kg of rat body weight), L-Arginine (100 mg/Kg) or saline. 10 minutes later, mediobasal part of hypothalamus (ie VMH+ARC, as described [54]) were collected, weighted (10 min delay was sufficient to cause significant functional effects, as attested by the IVGTT study) and homogenized in buffer (250 mM Tris-HCl, 10 mM EDTA and EGTA). NOS activity assay kit (Calbiochem, Darmstadt, Germany) based on the stoichiometric biochemical conversion of L-Arginine into L-Citrulline by NOS. Briefly tissues were incubated with radiolabelled [^3^H]-L-Arginine (1 µCi/µL) in a column with a resin which binds L-Arginine but not L-Citrulline, and we quantified [^3^H]-L-Citrulline radioactivity in the filtrate. To distinguish the constitutive NOS activity from iNOS activity, we omitted calcium chloride in the assay medium of the iNOS group. Results are given in CPM by mg of tissue.

### Carotid injections of pharmacologic NO pathway modulators

Prior to glucose tolerance test or during the euglycemic-hyperinsulinemic clamp, rats underwent an acute injection of L-NMMA into carotid catheter used for lipid infusion. L-NMMA (0.5 mg/kg of rat body weight), specific inhibitor of neuronal NOS, 7-Nitro-indazole (7-Ni, 0.5 mg/kg), specific inhibitor of inducible NOS, W1400 (0.05 mg/kg), L-Arginine, as the substrate of NOS (100 mg/kg) and Sodium Nitro-Prusside, SNP (0.5 mg/kg), as pharmacological NO donor (Sigma-Aldrich, L'Isle d'Abeau, France) was injected during 1 minute. Control injections were made with NaCl 0.9%.

### Glucose-induced insulin secretion test

Insulin secretion in response to glucose was studied in C and IL rats, with or without the injection of NO pathway modulators, 5 minutes before glucose injection. Food was removed 5 h before glucose injection into the saphenous vein (0.5 g/kg body weight) of anaesthetized rats. Blood samples were drawn from caudal vessels at 0, 1, 3, 5, 10, 15, 20 and 30 min following glucose injection. Glycemia was immediately measured using a glucose analyzer (Roche Diagnostics, Meylan, France). Plasma was then removed and stored at −20°C until radioimmunoassay of insulin.

### Measurement of glucose turnover rate during hyperinsulinemic-euglycemic clamp

After infusion, glucose turnover rate (GTR), ie both glucose disposal rate (GDR) and hepatic glucose production (HGP), was measured under hyperinsulinemic-euglycemic clamp conditions, in both IL rats and C rats before and after NO pathway modulators injection. Clamp was performed in 5 h food-deprived and anaesthetized rats. We previously reported that GTR values were similar in awake [55], [56] and anaesthetized rats [14], [57]. Blood sampling was performed with right jugular vein catheter. Basal blood glucose and plasma insulin were measured before insulin infusion. Then a priming dose of insulin (20 mU; Actrapid, Novo, Copenhagen, Denmark) and [^3^H]-3-glucose (4 µCi) was injected. After insulin (0.4 U.kg^−1^.h^−1^) and radiolabelled glucose (0.2 µCi/min) was continuously infused in sapheneous vein at a constant rate of 20 µL/min throughout the clamp study. During the procedure, blood was sampled from caudal vessels every 15 min to determine blood glucose and to adjust the rate of unlabelled glucose infusion to maintain euglycemia. The steady-state was attained within 30–40 min and maintained for 40 min thereafter. Blood samples were drawn during the steady-state euglycemic clamp for glucose disposal rate evaluation (t≈60, 70 and 80 min), before any injection of NO pathway modulator. After the carotid injection of L-NMMA, 7-Ni, Arginine and SNP, another set of blood was sampled when a new steady-state has been reached (t≈120 min after the beginning of the procedure).

For the assay of [^3^H]-3-glucose, blood samples were deproteinized (Ba(OH)_2_ and ZnSO_4_) and the supernatant evaporated to remove tritiated water. Dry residue was dissolved in 0.5 ml water and 9 ml scintillation solution was added (Aqualuma plus, Lumac, The Netherlands). Radioactivity was determined in a Packard Tri-Carb 460C liquid scintillation system.

### Calculations of glucose kinetics

In the basal steady state, the rate of glucose appearance (Ra, reflecting HGP) is equal to the glucose disposal rate (Rd, reflecting glucose utilization rate, GUR). During the euglycemic-hyperinsulinemic clamp, at steady state, Rd = Ra+Ra' (where Ra', GIR). Rd was calculated according to Rd = Ra = [^3^H]-3-glucose infusion rate (in disintegrations/minute) divided by blood glucose specific activity (in disintegrations/minute per milligram), Ra was obtained according to Ra = Rd−Ra', i.e. GUR-GIR.

### Hypothalamic nNOS and PIN protein expression

Mediobasal hypothalami were homogenized in 20 mmol/l Tris lysis buffer pH 7.4, and protease inhibitors (Roche Applied Science, Mannheim, Germany). Then, 50 µg soluble proteins were separated on a 7.5% SDS-polyacrylamide gel for nNOS detection and on a 14% tricine-polyacrylamide gel for PIN detection. Protein expressions were detected by Western Blot analysis. Monoclonal anti-nNOS and anti-PIN antibodies (Transduction Laboratories, Lexington, KY) were diluted at respectively 1∶750 and 1∶500. Immunoreactivity was detected by an enhanced chemiluminescence reaction (Amersham Biosciences, Little Chalfont, U.K) after incubation with a horseradish peroxidase-conjugated anti-mouse antibody (diluted 1∶3000, Sigma-Aldrich, Steinheim, Germany).

### Measurement of gene expression

Total RNA were isolated from hypothalamus using RNeasy Lipid mini kit (Qiagen, Courtaboeuf, France).Real time quantitative PCR amplification reaction were carried out in a LightCycler 1.5 detection system (Roche, Meylan, France) using the LightCycler FastStart DNA Master plus SYBR Green I kit (Roche). The mRNA transcript level for each gene was normalized against housekeeping gene cyclophilin B because we previously checked that this gene expression was not modified by lipid infusion.

### Systolic blood pressure measurement

To assess a potential effect of the 24 h lipid infusion on whole-body vascular tone, as well as a potential effect of carotid NOS inhibitor injection, we measured heart rate, systolic and diastolic blood pressure on both conscious IL and C rats before and after L-NMMA injection, using a computerized tail-cuff method (BP-2000, Visitech Systems, Apex, NC) [58]. Briefly animals were maintained restrained at 37°C in the dark and tail cuffs were placed on the tail of each of the rats. Animals were acclimatized with 10 preliminary measurements and the final systolic blood pressure and heart beat values were calculated as the average of 20 measurements.

### Analytical methods

Blood glucose was determined using a glucose analyzer (Accu-Chek, Roche, Mannheim, Germany). Plasma insulin concentration, as well as plasma corticosterone and ACTH levels, were determined by radioimmunoassay (DiaSorin, Les Ullis, France). FFA concentrations were measured using an enzymatic assay (NEFA-C test; Wako, Germany).

### Statistical analysis

Data are expressed as means±SEM. Data were analyzed using Stat View 5.0 for Windows (SAS Institute, Cary, NC) for statistical significance by applying respectively a Student t-test, a Mann-Whitney test or a two-way ANOVA test for repeated measurements, when appropriate. A p value of less than 0.05 was considered statistically significant.
